# Multilayer Films Based on Poly(lactic acid)/Gelatin Supplemented with Cellulose Nanocrystals and Antioxidant Extract from Almond Shell By-Product and Its Application on Hass Avocado Preservation

**DOI:** 10.3390/polym13213615

**Published:** 2021-10-20

**Authors:** Arantzazu Valdés, Carmen Martínez, Mari Carmen Garrigos, Alfonso Jimenez

**Affiliations:** Analytical Chemistry, Nutrition & Food Sciences Department, University of Alicante, P.O. Box 99, 03080 Alcante, Spain; cm_trinity@hotmail.com (C.M.); mc.garrigos@ua.es (M.C.G.); alfjimenez@ua.es (A.J.)

**Keywords:** almond shell by-product, antioxidant activity, reinforcing agent, active packaging, multilayer film, avocado preservation, poly(lactic acid), fish gelatin, corona treatment

## Abstract

In this work, poly(lactic acid) (PLA)/gelatin/PLA multilayer films supplemented with cellulose nanocrystals and antioxidant extract from almond shell (AS) by-products were developed by solvent casting technique for active food packaging. The almond shell antioxidant extract (ASE) was obtained by microwave-assisted extraction, while cellulose nanocrystals (CNCs) were extracted from AS by a sequential process of alkalization, acetylation and acid hydrolysis. Four formulations were obtained by adding 0 (control), 6 wt.% of ASE (FG/ASE), 4.5 wt.% of CNCs (FG/CNC) and 6 wt.% + 4.5 wt.% of ASE + CNCs, respectively, (FG/ASE + CNC) into fish gelatin (FG). PLA/FG/PLA multilayer films were prepared by stacking two outer PLA layers into a middle FG film. A surface modification of PLA by air atmospheric plasma treatment was optimized before multilayer development to improve PLA adhesion. Complete characterization of the multilayers underlined the FG/ASE + CNC formulation as a promising active reinforced packaging system for food preservation, with low values of transparency, lightness and whiteness index. A good adhesion and homogeneity of the multilayer system was obtained by SEM, and they also demonstrated low oxygen permeability (40.87 ± 5.20 cm^3^ mm m^−2^ day) and solubility (39.19 ± 0.16%) values, while mechanical properties were comparable with commercial plastic films. The developed multilayer films were applied to Hass avocado preservation. The initial degradation temperature (T_ini_), DSC parameters and in vitro antioxidant capacity of the films were in accordance with the low peroxide and anisidine values obtained from avocado pulp after packaging for 14 days at 4 °C. The developed PLA/FG/PLA films supplemented with 6 wt.% ASE+ 4.5 wt.% CNCs may be potential bioactive packaging systems for fat food preservation.

## 1. Introduction

The worldwide production of avocado showed an important increase from 3.8 to 7.2 M tones between 2009 and 2019. Nowadays, Mexico is the leading producer of avocados, representing nearly 32.0% of the worldwide production, followed by the Dominican Republic (9.2%), Peru (7.5%), Colombia (7.4%), Indonesia (6.4%) and Kenya (5.1%), among others [1]. Hass avocado is the most commercialized cultivar, accounting for approximately 80–95% of total harvested avocado [2]. About 15 wt.% of its composition corresponds to the fat fraction, containing up to 71% of monounsaturated fatty acids [3]. Avocados have a relatively short lifespan due to their climacteric nature and high content in unsaturated fatty acids, which make them prone to the lipid oxidation processes [2,4]. As a result, this fruit contributes to the 66% of vegetables and fruits going to waste annually [5]. To overcome this drawback, the development of novel active food packaging systems by the addition of agri-food by-products as new sources of natural antioxidant agents has been paid special attention to in recent years [6].

Almond shell (AS) is the lignocellulosic material of the almond husk, contributing around 35−75 wt.% of the total fruit weight [7]. Consequently, several million tons of almond shells are discarded annually. However, this by-product has been reported to be rich in bioactive compounds with interesting antioxidant and antiradical properties [8,9]. AS has also been considered as a rich source of cellulose nanocrystals (CNCs) due to its high amount of α-cellulose (near 40 wt.%), which could be used as reinforcement for polymers in food packaging applications [10].

Among the current polymer matrices, fish gelatin (FG) has been widely reported as a potential protein biopolymer due to its capacity to form films and for its flexibility, transparency and biodegradability [11]. In addition, the ability of FG to form intermolecular interactions due to the non-polar and polar amino acid components has allowed the development of effective active edible films [12]. However, the inherent hydrophilic nature of FG has limited its application in food packaging. This drawback can be overcome by laminating FG films with biodegradable polymers resistant to moisture in a multilayer structure. FG can be considered a good candidate for the development of sustainable multilayer systems combined with poly(lactic acid) (PLA), without compromising the sustainable nature of PLA. In addition, FG has higher barrier properties to gases compared to PLA in dry environments. Thus, the development of a PLA/FG/PLA laminate structure could protect the inner FG layer from the exposition to water molecules and increase the barrier properties of PLA [13]. Some authors have previously studied the development of PLA/FG/PLA three-layer systems [14,15,16,17]. However, to the best of our knowledge, no active multilayer packaging systems based on this combination have been reported in the literature.

The aim of this work was the development of active multilayer films based on PLA and FG for food packaging applications reinforced with CNCs supplemented with an antioxidant extract (ASE) obtained from AS by-products. Different concentrations of the studied additives were incorporated into the fish gelatin (FG) matrix: 0 (control); 6 wt.% of ASE (FG/ASE); 4.5 wt.% of CNCs (FG/CNC); and 6 wt.% + 4.5 wt.% of ASE + CNCs, respectively (FG/ASE + CNC). Multilayer films were prepared by stacking outer PLA layers, resulting in a PLA/FG/PLA structure. For this purpose, the following objectives were proposed in this work: (a) optimization of surface modification of PLA by air atmospheric plasma treatment; (b) development and characterization of multilayer PLA/FG/PLA films; (c) study of the in vitro antioxidant capacity of the developed active films; (d) evaluation of films efficiency for packaging of Hass avocado pulp up to 14 days at 4 °C in a shelf-life study.

## 2. Materials and Methods

### 2.1. Materials and Reagents

Commercial PLA films (Bio-FlexR F 2110) with a thickness of 15 µm and width of 300 mm were obtained from FKuR Kunststoff GmbH (Willich, Germany). This PLA material has previously been used to obtain multilayer films based on whey protein and PLA [18]. Cold deep-water fish (G2963A) was obtained from Healan Ingredients (York, UK).

Marcona almond shells (AS) were kindly supplied by “Sirvent Almendras S.A.” (Alicante, Spain). One-hundred grams of AS were washed with 500 mL of cold distilled water, followed by drying at ambient temperature for 12 h and further drying at 40 °C for 4 h. A fine AS powder was obtained by two consecutive grinding steps. Firstly, AS was placed in a domestic grinder (Fagor, Spain) for 15 s to reduce its initial size; a fine powder was then obtained with a high-speed rotor mill (Ultra Centrifugal Mill ZM 200, RETSCH, Haan, Germany) equipped with a 1 mm sized sieve. All chemicals and reagents were of analytical grade and were purchased from Sigma-Aldrich (Madrid, Spain).

### 2.2. Additives Preparation from AS By-Products

#### 2.2.1. Antioxidant Extract (ASE)

The extraction of antioxidant compounds from AS was performed by microwave-assisted extraction (MAE) using a flexiWAVE microwave oven (Milestone srl, Bergamo, Italy). An amount of 4.000 ± 0.001 g of homogenized AS powder was placed in a 100 mL quartz flask connected to a vapour condenser containing 60 mL of 70% (*v*/*v*) ethanol as the extraction solvent. The sample was stirred at 500 rpm at an extraction temperature of 80 °C for 57 min at pH 8. The obtained extract was centrifuged at 4500 rpm for 5 min and then passed through a 0.45 μm PVDF filter (Teknokroma, Barcelona, Spain). The solvent was then removed under vacuum at 40 °C in a rotary evaporator (R-300, Büchi Labortechnik AG, Flawil, Switzerland), which was followed by lyophilization (LyoQuest Plus, Telstar, Madrid, Spain) to obtain the dried AS extract (ASE).

#### 2.2.2. Cellulose Nanocrystals (CNCs)

In order to obtain CNCs from AS, different optimized specific chemical treatments were carried out in the following order: alkalization, acetylation and acid hydrolysis. Alkalization was performed by MAE in a flexiWAVE microwave oven (Milestone srl, Bergamo, Italy). An amount of 3.000 ± 0.001 g of homogenized AS powder was placed in a 100 mL quartz flask connected to a vapor condenser containing 60 mL of 9 wt.% NaOH at 95 °C for 20 min. The sample was subsequently filtrated through a glass microfiber filter until pH 7–8 of the liquid filtrate was obtained, using distilled water for washing. The sample was dried in an oven at 60 °C for 24 h. Acetylation was then carried out for 60 min using an acetic acid/nitric acid solution (6:1 *v*/*v*) at 100 °C with an AS/acid ratio of 0.6:14 (*w*/*v*). Finally, acid hydrolysis was performed for 30 min using 64 wt.% of sulphuric acid at 45 °C with an AS/acid ratio of 1:10 (*w*/*v*). The final product was dialyzed in deionized water for 4 days until it reached pH 4–5, and the suspension was then lyophilized (LyoQuest Plus, Telstar, Madrid, Spain) to obtain dried CNCs with an average size of 53 ± 3 nm, measured by transmission electron microscopy (TEM). Samples were measured at an accelerating voltage of 80 kV. A drop of nanocrystals solution (0.1 *w*/*v* %) was deposited on the surface of a Cu grid covered with a thin carbon film. The particle dimension was determined by digital image analysis (GATAN Digital Micrograph 1.80.70 para GMS 1.8.0). In order to study the dispersion stability of CNCs in aqueous suspension, the zeta potential of CNCs was measured, in triplicate, using a Zetasizer Nano ZS 3000 analyser (Malvern Instruments Ltd., Worcestershire, UK) for CNCs suspensions diluted to 0.1 wt.% with deionized water. In this study, the average value of zeta potential obtained for CNCs suspension in neutral water was –26 ± 3 mV, suggesting that the CNCs suspension has good stability because, according to the literature, agglomeration of nanocellulose will occur if the zeta potential value is within the range of –15 to 15 mV.

### 2.3. Films Preparation

#### 2.3.1. Fish Gelatin Films

Gelatin films were prepared by solvent casting at room temperature according to a previous work with some modifications [19]. FG (8 wt.%) was dissolved in distilled water under stirring for 20 min at 35 °C; 20 wt.% (based on FG weight) of glycerol was then added and stirred for 10 min. Four different formulations were obtained by adding 0, 6 wt.% of ASE, 4.5 wt.% of CNCs, and 6 wt.% + 4.5 wt.% of ASE + CNCs, respectively (based on FG weight), into the film solution. In order to avoid bubble formation, the final solutions were sonicated for 30 min and were cast onto Petri dishes for drying at 50% relative humidity (RH) and 23 ± 1 °C in a climate chamber (Dycometal, Barcelona, Spain) for 24 h.

#### 2.3.2. Air Atmospheric Plasma Treatment

Prior to the lamination of FG and PLA monolayer films to obtain the trilayer PLA/FG/PLA systems, a surface modification of PLA films was optimized in order to increase their surface tension and polarity to improve wettability and adhesion properties with the FG monolayer [20]. Corona discharge is considered an environmentally friendly method that allows only the modification of the topmost layers, but not the bulk material.

Only one face of the PLA films was treated with the corona discharge under industrial conditions (ambient air and 23 °C) [21] by using a BD-20ACV corona treater (Electro-Technic Products, Inc., Chicago, IL, USA) with a custom power-line filter and a transistorised generator (rated power: 220 V; output frequency: 4–5 MHz) transformer of rated voltage 10–48 kV. The 76 mm field effect electrode was passed back and forth approximately 6 mm above each bonding surface with a treatment surface of 0.011 m^2^. Five treatment times were studied (0, 20, 40, 60 and 80 s). After each treatment, samples were dried in a vacuum chamber at 23 °C and 80 mbar for 48 h. In order to select the optimal treatment time, all samples were evaluated by X-ray photoelectron spectroscopy analysis and surface tension.

##### X-ray Photoelectron Spectroscopy Analysis (XPS)

Analysis was conducted in a Bruker D8-Advance diffractometer (Madrid, Spain) with a Kristalloflex K 760-80F X-ray generator equipped with Cu Kα radiation source, operating at 45 kV and 30 mA, respectively, as the applied voltage and current. Diffraction patterns were recorded from 2 to 30° 2θ using a scanning rate of 1° min^−^^1^.

##### Surface Tension

The surface tension of films (γ_s_) and their polar (γ^p^_s_) and dispersive (γ^d^_s_) components were determined according to Rocca-Smith et al. [20]. Seven drops of water, diiodomethane, ethylene glycol, formamide or glycerol, having a volume of 1 μL, were deposited on the treated PLA film surface. Contact angle measurements were carried out at 23 ± 2 °C and ambient relative humidity of 50% in an SEO—Phoenix 300 Touch Automatic equipment (Kromtek, Malaysia).

#### 2.3.3. Trilayer PLA/FG/PLA Films

Multilayer films were prepared by stacking an outer PLA layer, an FG inner layer and a second outer PLA layer subjected to thermo-compression processing using an industrial Powerbond Modular Laminating Range (Reliant Machinery Inc., Philadelphia, PA, USA), without the addition of any adhesive between the layers. The lamination was carried out at 110 °C and compression of 4 MPa with a laminating belt speed of 3 m min^−^^1^, with a further cooling step at room temperature and atmospheric pressure. The obtained trilayer films were formulated as shown in Table 1, and they were stored at 25 ± 2 °C and 50 ± 2% of RH.

### 2.4. Multilayer Films Characterization

#### 2.4.1. Thickness, Transparency and Colour Values

Thickness and transparency values were measured using a 293 MDC-Lite Digimatic Micrometer (Mitutoyo, Japan) and a UV-Vis spectrophotometer (Spectronic BioMate 3, Thermo Electron Corporation, Warwickshire, UK), respectively, according to the methods described in a previous work [19].

The colour properties of the films were determined, in triplicate, with a Konica CM-3600d spectrophotometer (Konica Minolta Sensing Europe, Valencia, Spain) using the CIELAB colour notation system, according to Valdés et al. [22].

#### 2.4.2. ATR-FTIR Analysis

ATR-FTIR spectra were collected, in triplicate, using a Bruker Analitik IFS 66 FTIR spectrometer (Ettlingen, Germany) equipped with an ATR accessory. Films with a size of 1 × 1 mm^2^ were directly placed on the ATR crystal area. Spectra were recorded in the absorbance mode from 4000 to 600 cm^−1^, using 64 scans and 4 cm^−1^ resolution, and corrected against the background spectrum of air.

#### 2.4.3. Scanning Electron Microscopy (SEM)

Morphological characterization of the cryo-fractured surfaces of the films was performed using a JEOL JSM-840 microscope (Peabody, MA, USA) running at 10 kV. Samples were coated with gold under vacuum using an SCD 004 Balzers sputter coater (Bal Tec. AG, Fürstentum, Lichtenstein) prior to scanning.

#### 2.4.4. Thermal Analysis

Thermal characterization of the films was performed using thermogravimetric analysis (TGA). A TGA/SDTA 851 Mettler Toledo (Schwarzenbach, Switzerland) thermal analyser was used in this work. An amount of 4 mg of the sample was heated from 30 to 700 °C at a rate of 10 °C min^−1^ under nitrogen atmosphere (50 mL min^−1^). Two thermal parameters were determined, in triplicate: initial degradation temperature (T_ini_), calculated at 5% of weight loss, and temperature of maximum decomposition rate (T_max_).

A TA DSC Q-2000 instrument (New Castle, DE, USA) was used for differential scanning calorimetry (DSC) analysis to study the thermo-oxidative performance of films. An amount of 4.00 ± 0.01 mg of each sample was used. For oxidation onset temperature (OOT) determination, samples were heated up to 200 °C under oxygen atmosphere at a rate of 10 °C min^−^^1^. For oxidation induction time (OIT) determination, samples were heated up to 150 °C under nitrogen atmosphere at a rate of 100 °C min^−^^1^. Both parameters were obtained as described elsewhere [19].

#### 2.4.5. Mechanical Properties

Three tensile parameters (elastic modulus, tensile strength and elongation at break) were obtained from the stress–strain curves using a 3340 Series Single Column System Instron Instrument, LR30K model (Lloyd Instruments Ltd., Fareham Hants, UK), as described in a previous work [22].

#### 2.4.6. Barrier Properties

Oxygen transmission rate (OTR) tests were carried out with an oxygen permeation analyser (8500 model Systech Instruments, Metrotec S.A, Lezo, Spain) according to Valdés et al. [23], while the solubility of the films was determined, in triplicate, as previously detailed by Hosseini et al. [24].

#### 2.4.7. Antioxidant Activity and Total Phenolic Content (TPC) of Films

The evaluation of the in vitro antioxidant capacity of the films was performed using a method previously reported [22], with some modifications. Films with a size of 1.5 × 1.5 cm^2^ were extracted for 3 h with 12 mL of 96 wt.% ethanol at 25 ± 1 °C under 100 rpm in the absence of light. This extract was used to measure antioxidant activity using DPPH, FRAP and ABTS antioxidant assays, as well as total phenolic content (TPC), in triplicate, as reported by Valdés et al. [22].

### 2.5. Shelf-Life Study of Avocado Packaged in Multilayer Films

#### 2.5.1. Processing and Conditioning of Avocado Samples

An amount of 50 ± 1 g of crushed avocado pulp was placed in a Petri dish with 14 cm diameter and wrapped in the middle of two developed multilayer films, being in contact with the sample above and below. The sample was protected from oxygen through a hermetic closure during the shelf-life study. Samples were placed at 4 ± 1 °C for 14 days, ensuring a real conservation study of the food product.

#### 2.5.2. Oxidative Stability Study of Packaged Avocado

Three different times were studied, in triplicate, in order to evaluate the oxidative stability of the packaged sample: 0, 7 and 14 days. For each day, primary and secondary oxidation products were evaluated by determining the peroxide (PV) and p-anisidine values (AV), respectively. PV was determined following the ISO 3960 standard [25], while AV was measured using the IUPAC 2.504 method [26]. Avocado pulp was previously submitted to oil extraction by MAE using a flexiWAVE microwave oven (Milestone srl, Bergamo, Italy). An amount of 5.000 ± 0.001 g of homogenized avocado pulp was placed in a 100 mL quartz flask connected to a vapour condenser containing 60 mL of ethyl acetate as extraction the solvent [27]. The sample was stirred at 500 rpm at an extraction temperature of 65 °C for 30 min. The obtained oil was separated from the solid by vacuum filtration. The solvent was then removed under vacuum at 40 °C in a R-300, Büchi Labortechnik evaporator (AG, Flawil, Switzerland).

The pH of each sample was also measured, in triplicate. Tests were performed by potentiometric analysis using a pH-meter basic 20 (Crison Instruments, Barcelona, Spain). An amount of 5.000 ± 0.001 g of avocado pulp and 50 mL of distilled water was vortexed for 5 min before tests.

### 2.6. Statistical Analysis

Statistical analysis of experimental data was performed with SPSS commercial software (Version 15.0, Chicago, IL, USA). A one-way analysis of variance (ANOVA) was carried out. Differences between average values were assessed on the basis of confidence intervals using the Tukey test at a confidence level of 95% (*p* < 0.05).

## 3. Results

### 3.1. Surface Properties of PLA Films after Corona Treatment

Table 2 shows the results obtained for surface properties by XPS and goniometry. XPS analysis revealed significant differences (*p* < 0.05) on the PLA film surface with the different corona treatment times. In particular, the oxygen composition (%) tended to increase, whereas carbon composition decreased with increasing treatment time. As a consequence, an increase in the O/C ratio was found when the PLA surface was treated with corona discharge from 0 to 80 s, with values ranging from 0.18 ± 0.02 to 0.37 ± 0.02%, respectively. A similar increase was also observed in previous studies when treating PLA with atmospheric plasma treatments [20,28,29]. These findings give evidence that new oxygen-containing groups were created on the PLA surface after the corona treatment.

**Figure 1:**
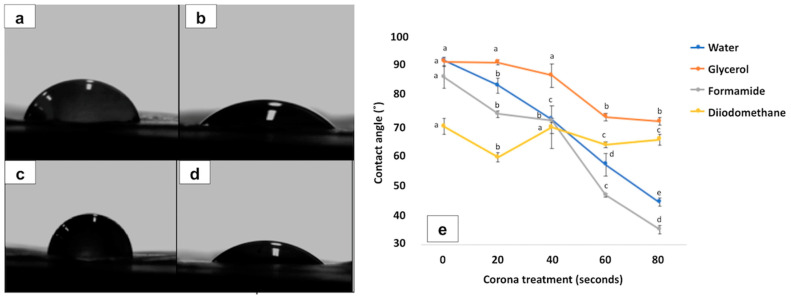
Variations in contact angle of PLA films after different corona treatments, obtained by goniometry: formamide drop shape for 0 (**a**) and 80 s (**b**); water drop shape for 0 (**c**) and 80 s (**d**); (**e**) contact angle values for different liquids at different corona treatments. Different letters (a,b,c,d,e) within the same time indicate statistically significant different values (*p* < 0.05).

Figure 1 shows the variations in contact angle of PLA films after different corona treatments obtained by goniometry. As an example, Figure 1a,b shows the behaviour of formamide drop shape after 0 and 80 s of corona treatment, respectively, while Figure 1c shows the behaviour against water after 0 s and Figure 1d after 80 s. Figure 1e shows the contact angle results obtained for PLA treated at different times with different liquids. As can be observed, 80 s of corona treatment allowed obtaining the lowest contact angle values for all tested liquids compared to the other times. The contact angle results demonstrated that PLA surfaces that had not been subjected to corona treatment showed higher values, while corona treatment for 80 s showed the lowest values, these being more noticeable for water. These results were in agreement with surface tension properties. As a result, the use of corona treatment was found to increase the polar contribution as well as the dispersive component, showing an overall surface tension increase (Table 2). These results are in line with previous reported studies [20,30,31,32], 80 s being the most suitable corona treatment time for the PLA surface, improving the adhesiveness when in contact with the intermediate fish gelatin monolayer.

### 3.2. Thickness, Transparency and Colour

**Figure 2:**
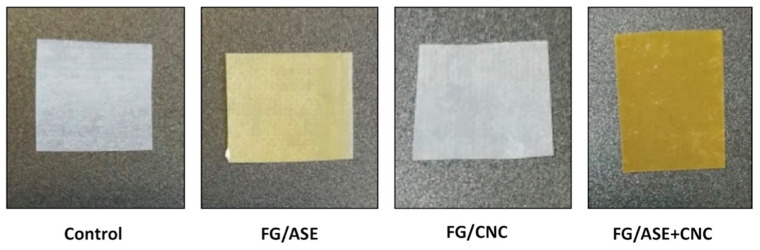
Visual appearance of the obtained multilayer films.

The visual appearance of the obtained multilayer films is shown in Figure 2. All films showed a high visual homogeneity. No significant differences (*p* > 0.05) were obtained regarding thickness values for samples (Table 3), suggesting that both ASE or CNCs addition did not substantially affect the film formation process [12]. However, the addition of the studied additives to FG induced some changes in the multilayer films’ transparency and colour properties (Table 3). Indeed, transparency values to visible radiation (%) of films decreased from 7.89 ± 0.36 for the control to 6.67 ± 0.55 for FG/CNC, followed by 5.92 ± 0.15 and 5.62 ± 0.12 for FG/ASE and FG/ASE + CNC formulations, respectively. This behaviour could be related to the colour properties obtained for the multilayer films. In addition, the low transparency of the multilayer films might be related to the ordered arrangement of PLA molecules in the external layers due to the partial crystallization and the compact structure of the semi-crystalline PLA in the film matrix [14]. Regarding colour properties, control and FG/CNC formulations showed slight differences in L*, a*, b* and whiteness index (WI) parameters. However, a significant (*p* < 0.05) decrease in L* (lightness) and WI values and an increase in a* and b* parameters was obtained for FG/ASE and FG/ASE + CNC films (Table 3). These results highlighted the lower transparency of these polymer systems in contrast to the control and FG/CNC formulations, which might be helpful in the prevention of the oxidative deterioration of packaged foodstuffs [22].

Concerning total colour difference (ΔE), a clearly significant (*p* < 0.05) increase was observed when ASE was added to the multilayer film, being higher for films that included the combined effect of ASE and CNCs due to the natural yellowish colour of the extract. Similar results were recently reported for PCL-based films loaded with an almond skin extract [33]. In general terms, the neat visual appearance and low transparency of the studied multilayer films could be adequate for food packaging applications such as trays or containers.

### 3.3. ATR-FTIR Analysis

**Figure 3:**
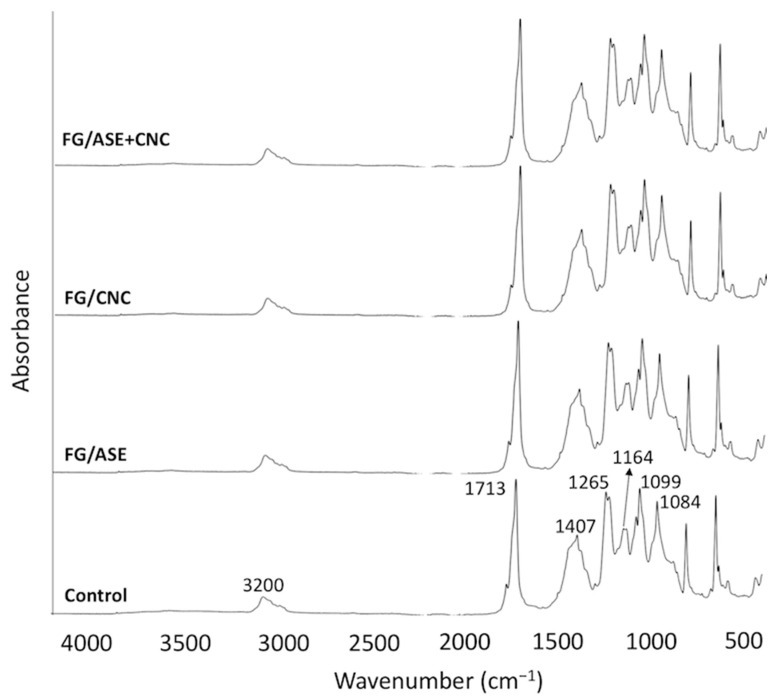
ATR-FTIR spectra of the obtained multilayer films.

The chemical structure of the obtained films was investigated by ATR-FTIR analysis (Figure 3). All samples showed the characteristic peaks of the PLA layer [14,15]. The bands at 3200 cm^−1^ were assigned to −CH stretching. The typical asymmetric stretching of the carbonyl group (C=O) in the PLA film was attributed to lactide at 1713 cm^−1^. On the other hand, a sharp peak centred at 1407 cm^−1^ was assigned to the lactides −CH_3_ group. The symmetric –CH_3_ deformation vibrational peaks were observed at 1265 cm^−1^. In addition, the symmetric C–O–C stretching peak of the PLA ester group was observed at 1164 cm^−1^. Finally, the peaks detected at 1099 and 1084 cm^−1^ were attributed to the stretching vibration of –C–O or –C–OH deformation vibration. No significant differences (*p* > 0.05) were observed in wavenumber and absorbance values for all the detected bands among the studied samples. This fact could be explained by considering the fact that the ATR-FTIR technique analyses the films surface. In this work, PLA served as the outer layer of the multi-layered PLA/FG/PLA films. Therefore, it was expected that the FTIR spectra of all samples showed the characteristic peaks of the PLA layer, as previously reported for different multilayer systems based on a PLA/FG/PLA structure [14,15].

### 3.4. Morphological Characterization by SEM

**Figure 4:**
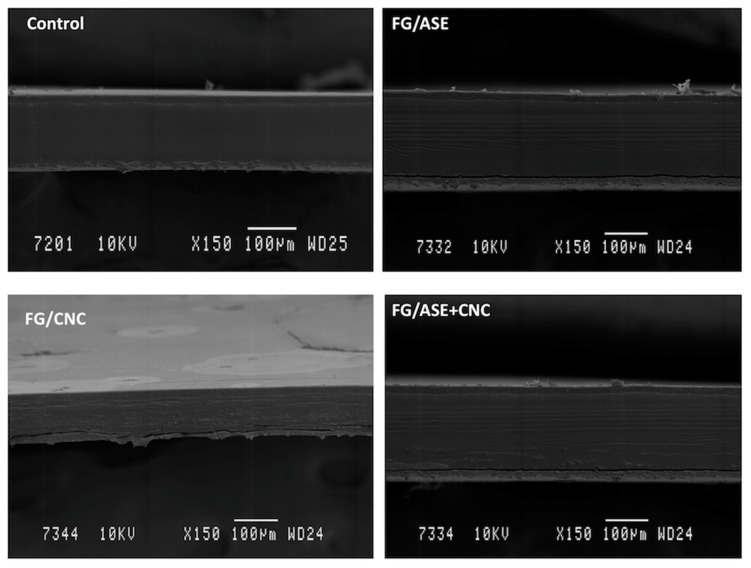
Cross-sectional SEM images of multilayer films.

SEM micrographs of the cryo-fractured cross section of multilayer films are shown in Figure 4. SEM images clearly show three different layers, corresponding to the upper and lower PLA layers and the middle FG layer. In all formulations, the PLA layers showed a homogeneous and smooth surface without any pores. In addition, the control FG layer exhibited a uniform structure without the presence of cracks or bubbles. Similar results were reported for multilayer film structures based on PLA and FG prepared using the solvent casting technique [14,15]. FG/ASE and FG/ASE + CNC films showed some isolated spots, which were related to the addition of ASE, compared to the control film, whereas the FG/CNC formulation exhibited some roughness on the FG layer, although no significant structural modifications were observed. It should be highlighted that the CNCs used in this work showed a zeta potential value of –26 ± 3 mV, suggesting a good dispersion of these CNCs in hydrophilic matrices such as fish gelatin [34]. In this sense, zeta potential values close to –30 mV indicate a good stability of the prepared dispersion and aggregation resistance [35].

On the other hand, previous studies have reported that the addition of active agents extracted from agricultural residues into different polymer matrices could decrease the homogeneity of the polymer layers due to the hydrophilicity of the polyphenolic compounds present in the active extracts. In our study, the phenolic compounds present in ASE could interact with the protein chains of FG and cause partial protein aggregation. Similar microstructural findings were reported for poly(ε-caprolactone)-based films containing an almond shell extract [33], edible films gelatin-based with oregano extract [36] and tilapia skin gelatin incorporated with an ethanolic extract from coconut husk [37]. In a different study, the addition of microcrystalline cellulose into a fish gelatin film was reported [38], showing cross-section SEM micrographs surface roughness in all composite films when microcrystalline cellulose was added. This behaviour was attributed to some excessive microcrystalline cellulose overlapping with each other during the film formation process.

After performing the drying process in the casting technique, water was evaporated from the film, resulting in a small number of holes and gaps. Nevertheless, no separation or voids between the PLA layers and the inner gelatin layer was noticeable (Figure 4), and homogeneous multilayer films were obtained. Accordingly, these results proved the satisfactory effect of the corona treatment and lamination process used to obtain the multilayer systems studied in this work.

### 3.5. Thermal Characterization

**Figure 5:**
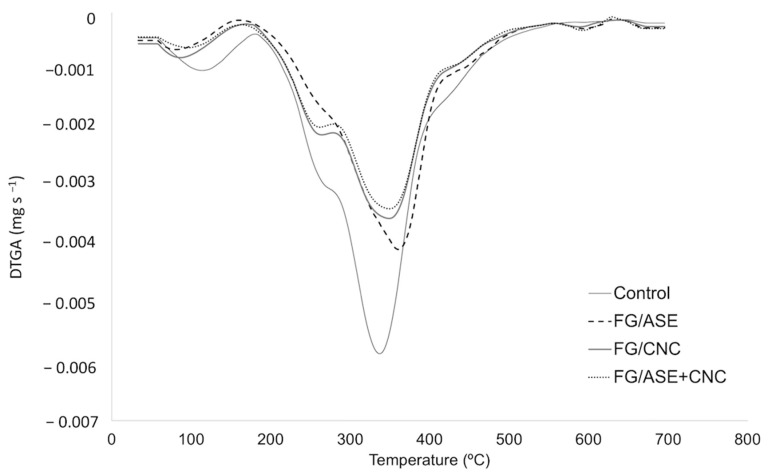
DTG curves for control, FG/ASE, FG/CNC and FG/ASE + CNC films in nitrogen atmosphere (10 °C min^−1^).

Thermal decomposition of film samples was studied by TGA in order to evaluate the effect of ASE and CNCs addition into the FG interlayer on the thermal stability of the obtained multilayer systems. The DTG curves of the samples are shown in Figure 5. All films showed one first degradation peak in the range of 50–160 °C, which was related to the loss of adsorbed and bound water in the films [15]. The main degradation step (T_max_) was associated with the decomposition of the main components present in the film matrix, in agreement with values formerly reported for multilayer systems based on PLA and FG [14,15]. Despite ASE or CNCs addition, the greater thermal resistance of the obtained multilayer systems might be ascribed to the PLA layers and their high intermolecular interaction and compact network, as well as to the presence of crystalline structures in the films [14]. According to T_ini_ values, the FG/ASE + CNC film showed the highest thermal resistance, followed by the FG/ASE film, compared to the control and FG/CNC formulations (Table 4). It could therefore be concluded that the addition of ASE significantly increased the thermal stability of the polymer matrix. This behaviour was more noticeable when ASE was combined with CNCs (Table 4).

The thermo-oxidative resistance of multilayer films was evaluated by the determination of DSC parameters, and they are reported in Table 5. No significant differences (*p* > 0.05) in OOT values were observed between the control and FG/CNC films; whereas significant (*p* < 0.05) higher values were obtained for the FG/ASE formulation, followed by the FG/ASE + CNC multilayer film. A similar trend was observed for OIT values, showing that the control film had the lowest value, followed by the FG/CNC, FG/ASE and, finally, the FG/ASE + CNC films. These results are in line with T_ini_ values obtained by TGA (Table 4), suggesting the potential of ASE and CNCs simultaneous addition to improve the thermo-oxidative resistance of PLA/FG/PLA films.

### 3.6. Mechanical Properties

Three tensile parameters (elastic modulus, tensile strength and elongation at break) were evaluated in the studied multilayer films (Table 4). Tensile strength and elongation at break values of the control film were 23.69 ± 0.48 MPa and 3.26 ± 0.16%, respectively, which were in good agreement with previously reported values for PLA/FG/PLA films [14,15,16]. The tensile strength values of multilayer films were comparable to those reported for different widely used plastic films, such as HDPE (17.9–33.1 MPa), LDPE (15.2–78.6 MPa) and polypropylene (20–152 MPa) [15].

The presence of ASE in the gelatin interlayer directly affected the mechanical properties of the multilayer films, resulting in an increase in the three studied mechanical parameters compared to the control film. This fact suggested a good dispersion of the active extract into the FG layer in the FG/ASE and FG/ASE + CNC formulations, reducing the occurrence of stress concentration centres. Similar effects were observed in films based on FG supplemented with *Amaranthus* leaf extract [39]; aqueous extracts from rowanberry, blue-berried honeysuckle and chokeberry pomace [40] and seaweed extract [41]. The polyphenolic compounds present in the active extracts could form hydrogen and covalent (non-disulphide) bonds with the amino and hydroxyl groups of polypeptides in gelatin. As a result, a decrease in protein–protein interactions in the film network could take place [42,43]. Thus, ASE addition seems to weaken the intermolecular interaction between gelatin and glycerol, resulting in an increase in the ductility of films [44,45].

The presence of CNCs into the FG layer evidenced the typical results obtained in reinforced polymer matrices regarding elastic modulus and elongation at break values. The FG/CNC film showed a decrease in elongation at break and an increase in elastic modulus compared to the control film when CNCs obtained from almond shell were added at 4.5 wt.% loading. Similar results were reported for FG films containing di-aldehyde cellulose nanocrystals at 15 wt.% [46]. However, a noticeable decrease in tensile strength for the FG/CNC film was observed in contrast to the control film (Table 4). This fact could be due to some CNCs agglomeration into the FG matrix, affecting the structure order [47], in accordance with SEM micrographs obtained for the FG/CNC formulation. As a result, the cohesion of the interlayer matrix and resistance to rupture were reduced in the multilayer film [38,48,49]. Similar results were obtained for Mesquite seed gum and tilapia FG composite films supplemented with CNCs at different concentrations [34]. In this work, elongation at break and tensile strength values noticeably decreased by the addition of CNCs at 5 wt.%. Some authors have attributed this behaviour to the rigidity of CNCs and the interactions between them to a reduction in the plasticizing effect of glycerol. This phenomenon was not observed for the FG/ASE + CNC formulation, which showed an increase in tensile strength and elastic modulus compared to the control film, together with a slight increase in the elongation at break, suggesting some reinforcement effect, probably due to the interaction of ASE with CNCs, glycerol and the FG matrix, as previously explained. The presence of CNCs allowed the establishment of interactions through hydrogen bonding with the polymer components, mainly due to hydroxyl groups present in CNCs and the presence of polyphenolic compounds in ASE [50]. Concerning tensile strength, slightly significant differences (*p* < 0.05) were obtained between the FG/ASE and FG/ASE + CNC multilayer systems as a consequence of the presence of CNCs.

### 3.7. Barrier Properties

The OTR and solubility results of the studied multilayer films are shown in Table 4. Multilayer films showed OTR values ranging from 3.04 to 57.11 cm^3^ mm m^−2^ day. The highest value was obtained for the FG/ASE film, followed by the FG/ASE + CNC, control and, finally, the FG/CNC formulations. Similar OTR values were reported for PLA/FG/PLA trilayer systems [15]. The enhanced oxygen barrier properties of the multilayer structure compared to the PLA outer layers may presumably be linked to the impermeable FG interlayer on the polymer substrate, retarding oxygen movement through the film [51]. These values are comparable to those reported for some commercial packaging materials which are considered good oxygen-barrier materials, such as polyethylene terephthalate (1–5 cm^3^ mm m^−2^ day), polypropylene (50–100 cm^3^ mm m^−2^ day), polystyrene (100–150 cm^3^ mm m^−2^ day) and polyethylene (50–200 cm^3^ mm m^−2^ day) [52].

In general, all films showed solubility values near or below 50%, being suitable for fat food packaging applications, as these foodstuffs present a lower water content in their composition. The highest solubility value was obtained for the FG/ASE film, followed by the control and, finally, the FG/ASE + CNC and FG/CNC formulations. As expected, the addition of ASE (FG/ASE film) increased OTR and solubility values compared to the FG/CNC film, which showed considerably decreased values, in particular for OTR. The strong hydrogen bonding between CNCs could make them act as nucleating agents, increasing the tortuosity of the diffusion pathway, contributing to improve oxygen barrier properties [53,54]. The combined addition of ASE and CNCs in films decreased OTR and solubility values in the FG/ASE + CNC film compared to the FG/ASE film due to protein-phenolic-CNCs-interactions in the polymer matrix, reducing the availability of the hydroxyl and amino groups’ interactions with water. As a result, the tortuosity of the polymer matrix increased, decreasing water solubility [55].

### 3.8. Antioxidant Activity of Films

The control and FG/CNC films showed non-detectable in vitro antioxidant activity using the DPPH, ABTS and FRAP methods, due to the absence of polyphenolic compounds, as TPC results underlined. As a result, only the FG/ASE and FG/ASE + CNC formulations showed in vitro antioxidant capacity by all tested methods, being in all cases higher for the FG/ASE + CNC multilayer. This behaviour was attributed to the antioxidant effect of the active compounds present in the MAE extract and the structural effect of CNCs, which allowed greater migration of antioxidant compounds from the polymer matrix due to interactions between components inside the matrix, as previously described in the mechanical and barrier properties sections. The antioxidant capacity of ASE could be related to its phenolic compound content, chlorogenic acid and catechin being the most abundant polyphenols, followed by protocatechin acid, caffeic acid, epicatechin, p-coumaric acid and quercetin-3-glucoside, according to previous reported studies [8,56,57]. A high number of phenolic-OH groups can be found in these compounds, with potential hydrogen-atom transfer sites, which can be related to their antioxidant capacity. On the other hand, it has been reported that active compounds could be released from the polymer matrix by diffusion through the expanded structure [58]. The FG/ASE + CNC multilayer film showed significantly (*p* < 0.05) higher antioxidant activity compared to the FG/ASE film, which could be attributed to the chemical crosslinking degree of this film. The FG/ASE cross-linking could lead to a lower free ASE content in the films and, consequently, a lower hydrogen donor ability, thus reducing the antioxidant activity compared to the FG/ASE + CNC film. However, the antioxidant effect was substantially increased with CNCs addition because CNCs and gelatin interactions can decrease the gelatin accessibility to ASE. Similar results were observed for gelatin films incorporating cellulose nanocrystals and tannic acid [59].

### 3.9. Oxidative Stability Study of Packaged Avocado in Multilayer Films

**Figure 6:**
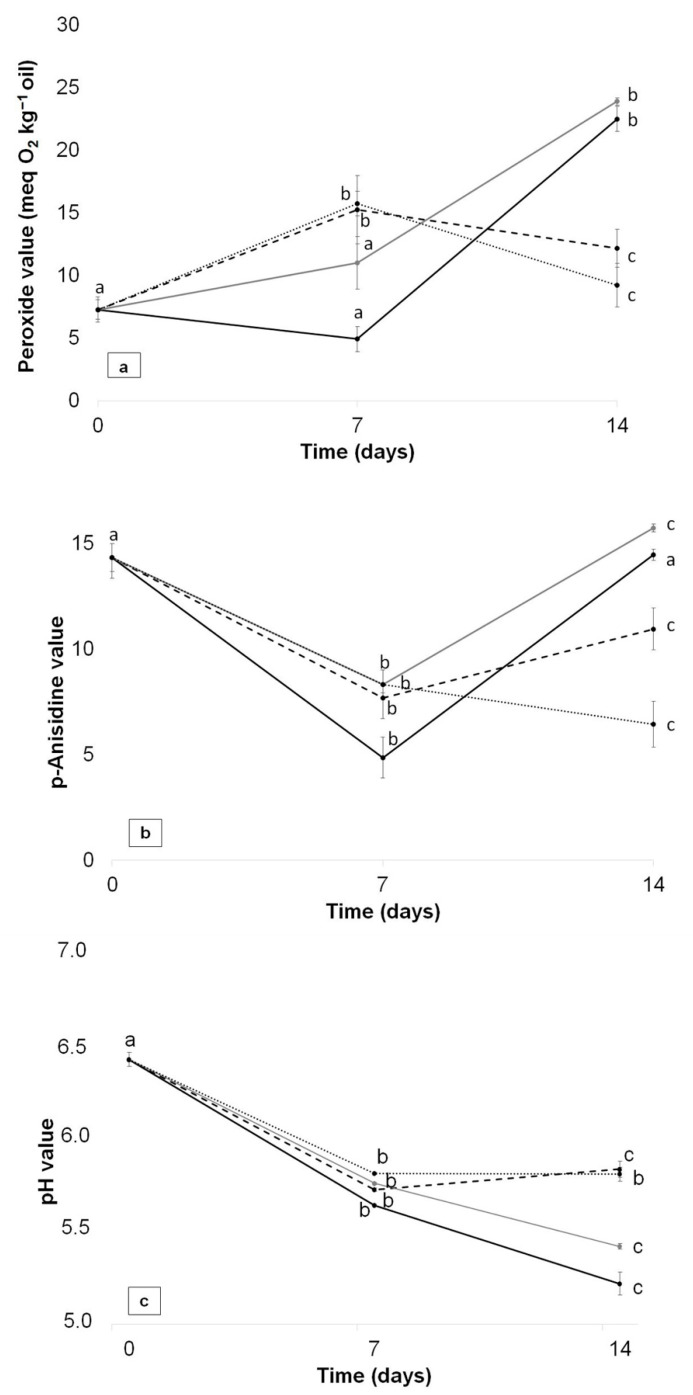
Changes in PV (**a**), AV (**b**) and pH (**c**) for control (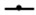), FG/ASE (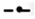), FG/CNC (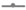) and FG/ASE + CNC (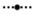) films as a function of time at 4 °C. Mean ± SD, *n* = 3. Different letters (a,b,c) within the same multilayer film and parameter indicate statistically significant different values (*p* < 0.05).

The oxidative stability of packaged avocado samples in the multilayer films was evaluated at 0, 7 and 14 days of study by determining PV, AV and pH values in avocado oil. The changes observed in these parameters for avocado oil during storage in the multilayer films are shown in Figure 6. The initial PV value (day 0) of avocado oil was low (7.3 ± 0.8 meq O_2_ kg oil), being indicative of a non-advanced initial state of oxidation. Similar values were reported for avocado Hass cultivar [4,60,61]. After 14 days of storage, all samples showed an increased in PV values, and this effect was mainly attributed to the high degree of unsaturation present in avocado oil, among other factors, because unsaturated fatty acids generally oxidize faster than saturated fatty acids, even under refrigeration or freezing [4]. Hass avocado oil has been reported to be rich in oleic, arachidonic and palmitic acids, together with vitamin E, phytosterols, chlorophylls and carotenoids [62]. In order to prevent the oxidation of avocado during storage, active packaging was proposed in the present work.

Figure 6a shows PV values of avocado samples packaged into the different developed multilayer films. A continuous increase in PV values with packaged time was observed for the control and the FG/CNC films as a consequence of hydroperoxides formation, reaching a maximum PV value after 14 days. These results may indicate that primary oxidation products formed in the early stages of the oxidation process would have given way to secondary oxidation products. A different behaviour was observed for the FG/ASE and FG/ASE + CNC films, which showed an initial increase in PV values with time, reaching a maximum after 7 days of study and a final decrease at 14 days, appearing to be more resistant to oxidation. In this case, hydroperoxides may appear only transitorily and rapidly decompose into volatile and non-volatile products after 14 days [63]. As can be seen, at the end of the study (14 days), the lowest PV value was obtained for the FG/ASE + CNC film (9.2 ± 1.5 meq O_2_ kg^−^^1^ oil), followed by FG/ASE (12.1 ± 1.0 meq O_2_ kg^−^^1^ oil) and, finally, the control (22.5 ± 1.6 meq O_2_ kg^−^^1^ oil) and FG/CNC (23.9 ± 2.1 meq O_2_ kg^−^^1^ oil) formulations showing no significant differences (*p* > 0.05) between them. As a consequence, it was concluded that the FG/ASE + CNC film showed the highest antioxidant activity, being in accordance with T_ini_ and DSC parameters and the in vitro antioxidant activity results previously discussed.

In the second phase of the oxidation process, the primary products of oxidation, peroxides, decomposed and developed volatile substances such as aldehydes, which are responsible for the rancid smell and taste, and they can be evaluated by the determination of AV [64]. The initial AV value (day 0) of avocado oil was 14.3 ± 0.7, in accordance with values reported for the avocado oils of three Indonesian cultivars [64]. As can be seen in Figure 6b, avocado packaged into the control and FG/CNC multilayer films showed a similar behaviour to that observed in PV, showing a decrease after 7 days of storage followed by a final increase at 14 days, indicating a simultaneous formation of hydroperoxides and secondary oxidation compounds. Due to the low formation of primary oxidation products in the FG/ASE and FG/ASE + CNC formulations, AV tended to decrease with storage time. At the end of the study, all samples showed significant differences (*p* < 0.05). The lowest AV value was obtained for the FG/ASE + CNC film (6.4 ± 0.3), followed by the FG/ASE (10.9 ± 0.9), control (14.5 ± 0.3) and, finally, the FG/CNC (15.7 ± 0.2) films, in accordance with PV results.

The pH values of samples were also determined, and the results are shown in Figure 6c. Avocado fruit (cv. Hass) has a characteristic pH value close to neutral. The initial pH value (day 0) of avocado pulp was 6.41 ± 0.04, in accordance with values reported for avocado pulp of Hass cultivar [65]. A general decrease in pH values in all samples was observed with time. Some authors have reported that pH values can affect food quality parameters, especially colour, if decreasing lower than a value of 5 [66], which was not the case in this work. At the end of the study, these samples showed significant differences (*p* < 0.05). The highest pH value was obtained for the FG/ASE + CNC (5.80 ± 0.04) and FG/ASE (5.83 ± 0.04) films, showing no significant differences (*p* > 0.05) between them, followed by the FG/CNC (5.42 ± 0.02) and, finally, the control (5.22 ± 0.06) films. Thus, it was concluded that FG/ASE + CNC and FG/ASE formulations allowed better conservation of avocado samples with time.

## 4. Conclusions

Active films based on an PLA/FG/PLA multilayer structure supplemented with ASE and CNCs extracted from AS by-products were successfully developed. The surface modification of PLA layers by air atmospheric plasma treatment for 80 s prior to the lamination process improved their wettability and adhesiveness with the intermediate FG monolayer. The visual appearance and low transparency of the studied multilayers underlined their potential application for food packaging applications, such as in trays or containers. In addition, the low lightness (L*) and WI values obtained with the addition of ASE might be helpful to prevent oxidative deterioration of packaged foodstuff. SEM, mechanical and barrier properties underlined the interactions between FG, glycerol, ASE and CNCs and their impact on multilayer systems. All samples showed tensile strength values comparable to commercial plastics, low oxygen permeability values and solubility near or lower than 50%, which are desirable properties for food packaging applications, particularly for fat food with a low water content in their composition. The addition of ASE increased the ductility of the film due to hydrogen and covalent (non-disulphide) bonds between polyphenolic compounds and amino and hydroxyl groups of polypeptides in gelatin. The addition of CNCs reduced the resistance to rupture of the multilayer film due to the rigidity of CNCs and the interactions between them. However, the presence of hydroxyl groups in CNCs, combined with the polyphenolic moieties of ASE, allowed interactions through hydrogen bonding with the polymer matrix, suggesting some reinforcement effect in the FG/ASE + CNC film compared to the control film. The combination of ASE and CNCs also decreased OTR and solubility in FG/ASE + CNC formulation in contrast to the FG/ASE film due to protein–phenolic–CNCs interactions, increasing the tortuosity of the film with an improvement in barrier properties. The T_ini_, DSC parameters and in vitro antioxidant capacity of films underlined the potential of the combined effect of ASE and CNCs, suggesting FG/ASE + CNC film as a good antioxidant material. These results were in accordance with those found in the oxidative stability study of packaged avocado, obtaining low PV and AV values after 14 days of treatment. The obtained pH values suggested a better conservation of avocado samples with storage time. In conclusion, antioxidant films based on PLA/FG/PLA supplemented with ASE at 6 wt.% and CNCs at 4.5 wt.% have been shown to be a promising active reinforced packaging system for food preservation, also contributing to valorise AS by-products into a circular economy approach. In this line, an MAE procedure was proposed in the present work for ASE and CNCs extraction steps, showing several advantages over conventional extraction techniques, such as the reduction of energy consumption and solvent used for extraction, good reproducibility, and reduced extraction time with minimal sample manipulation for the extraction process. Acid hydrolysis is the most widely employed methods for obtaining CNCs; however, this procedure has some drawbacks related to economic and environmental aspects such as the energy demand of the process and the high amount of water and time required in the neutralization steps. Another possibility for producing CNCs could be the use of enzymatic hydrolysis, a promising environmentally friendly and sustainable route [66].

Finally, this work proposed the processing of the inner layer by the wet process of casting that needs solvents for the solution and dispersion of the polymer onto a flat surface; this is followed by drying in controlled conditions for the removal of the solvent and the formation of the film. This is a high-energy-consuming procedure, adequate for laboratories but not for the industrial scale-up. Thus, further work will be needed to produce the inner layer by the dry methods conventionally used in the food packaging industry, which includes extrusion, injection, blow-moulding, and heat-pressing processes.

## Figures and Tables

**Table 1:** Multilayer film formulations obtained in this work.

Formulation	Codification	Content (wt.%)			
		FG	Glycerol	ASE	CNCs
PLA/FG/PLA	Control	8	20	0	0
PLA/FG + ASE/PLA	FG/ASE	8	20	6	0
PLA/FG + CNCs/PLA	FG/CNC	8	20	0	4.5
PLA/FG + ASE + CNCs/PLA	FG/ASE + CNC	8	20	6	4.5

**Table 2:** Surface properties of PLA films after different corona treatment times. Mean ± SD. Different superscripts (a,b,c) within the same column indicate statistically significant different values (*p* < 0.05).

PLA Surface Treatment (s)	Elemental Composition (%) ^1^			Surface Tension (mN m^−1^) ^2^		
	C	O	O/C	γ^p^_s_	γ^d^_s_	γ_s_
0	85 ± 1 ^a^	15 ± 1 ^a^	0.18 ± 0.02 ^a^	3.1	17.9	21.0
20	81 ± 3 ^a,b^	19 ± 3 ^a,b^	0.24 ± 0.05 ^a,b^	4.3	21.9	26.3
40	78 ± 1 ^b^	22 ± 1 ^b^	0.28 ± 0.02 ^b^	11.8	16.2	27.9
60	79 ± 2 ^b^	21 ± 1 ^b^	0.27 ± 0.06 ^b^	18.9	21.4	40.3
80	73 ± 1 ^c^	27 ± 1 ^c^	0.37 ± 0.02 ^c^	26.5	20.5	47.1

C = carbon, O = oxygen, γ^p^_s_ = polar contribution, γ^d^_s_ = dispersive contribution, and γ_s_ = surface tension of the tested film. ^1^ Determined by XPS analysis. ^2^ Determined by the Owens-Wendt method.

**Table 3:** Thickness (*n* = 5), transparency and colour (*n* = 3) parameters obtained for multilayer films (Mean ± SD).

Parameter	Control	FG/ASE	FG/CNC	FG/ASE + CNC
Thickness (mm)	0.105 ± 0.011 ^a^	0.109 ± 0.008 ^a^	0.105 ± 0.004 ^a^	0.118 ± 0.014 ^a^
Transparency (%)	7.89 ± 0.36 ^a^	5.92 ± 0.15 ^b^	6.67 ± 0.55^c^	5.62 ± 0.12 ^b^
L*	83.95 ± 0.02 ^a^	60.09 ± 0.02 ^b^	83.94 ± 0.06 ^a^	50.21 ± 0.02 ^c^
a*	−2.58 ± 0.03 ^a^	10.35 ± 0.04 ^b^	−2.65 ± 0.29 ^a^	14.28 ± 0.02 ^c^
b*	5.23 ± 0.02 ^a^	36.77 ± 0.04 ^b^	8.85 ± 0.02 ^c^	31.93 ± 0.05 ^d^
ΔE	-	41.60 ± 0.05 ^a^	3.61 ± 0.04 ^b^	46.03 ± 0.29 ^c^
WI	83.75 ± 0.02 ^a^	46.38 ± 0.06 ^b^	83.43 ± 0.26 ^a^	36.31 ± 0.44 ^c^

Different superscripts (a,b,c,d) within the same row and parameter indicate statistically significant different values (*p* < 0.05).

**Table 4:** TGA parameters (*n* = 3), OOT and OIT (°C), obtained by DSC (*n* = 3), mechanical (*n* = 5) and barrier (*n* = 3) parameters of the studied multilayer films (Mean ± SD).

Parameter	Control	FG/ASE	FG/CNC	FG/ASE + CNC
T_ini_ (°C)	243 ± 2 ^a^	249 ± 1 ^b^	245 ± 2 ^a^	256 ± 1 ^c^
T_max_ (°C)	364 ± 4 ^a^	366 ± 2 ^a^	365 ± 3 ^a^	371 ± 3 ^a^
OOT (°C)	122 ± 3 ^a^	132 ± 1 ^b^	124 ± 1 ^a^	144 ± 1 ^c^
OIT (°C)	11.43 ± 0.27 ^a^	16.81 ± 0.35 ^b^	12.74 ± 0.27 ^c^	20.92 ± 0.64 ^d^
Tensile strength (MPa)	23.69 ± 0.48 ^a^	36.71 ± 0.11 ^b^	10.48 ± 0.03 ^c^	34.34 ± 0.02 ^d^
Elastic modulus (MPa)	6.33 ± 0.59 ^a^	8.63 ± 0.81 ^b^	9.77 ± 0.05 ^c^	8.54 ± 0.51 ^b^
Elongation at break (%)	3.26 ± 0.16 ^a^	4.60 ± 0.02 ^b^	0.86 ± 0.29 ^c^	4.69 ± 0.05 ^b^
OTR.e (cm^3^ mm m^−2^ day)	14.10 ± 0.86 ^a^	57.11 ± 4.73 ^b^	3.04 ± 0.18 ^c^	40.87 ± 5.20 ^d^
Solubility (%)	43.41 ± 2.68 ^a^	52.62 ± 1.25 ^b^	36.69 ± 2.95 ^c^	39.19 ± 0.16 ^c^

Different superscripts (a,b,c,d) within the same row and parameter indicate statistically significant different values (*p* < 0.05).

**Table 5:** DPPH results expressed as RSA (%) and ABTS, FRAP and TPC results expressed as µg of gallic acid equivalents (GAE) per gram of film (Mean ± SD; *n* = 3). nd = non-detected.

Antioxidant Essay	Control	FG/ASE	FG/CNC	FG/ASE + CNC
DPPH	nd	17 ± 1 ^a^	nd	28 ± 3 ^b^
ABTS	nd	268 ± 26 ^a^	nd	377 ± 15 ^b^
FRAP	nd	258 ± 1 ^a^	nd	409 ± 4 ^b^
TPC	nd	361 ± 9 ^a^	nd	566 ± 15 ^b^

Different superscripts (a,b) within the same row and parameter indicate statistically significant different values (*p* < 0.05).

## Data Availability

Not applicable.
